# Supplementary material for: In Silico Analysis of miRNA-Regulated Pathways in Spinocerebellar Ataxia Type 7
Source: Curr Issues Mol Biol. 2025 Mar 2;47(3):170. doi: 10.3390/cimb47030170 (PMC11941346; doi:10.3390/cimb47030170)
Supplement: Supplementary file 1 [file cimb-47-00170-s001.zip › cimb-3431946-supplementary.pdf]

Supplementary Table S1. Annotation of KEGG pathway enrichment and number of miRNA-regulated genes. The analysis was performed by applying conventional enrichment algorithms of hypergeometric test to the target genes. The number of hits indicates the miRNAs-regulated genes associated with each specific KEGG pathway. The adjusted p-value accounts for multiple comparisons and helps reduce the likelihood of false positives. The data was derived from miRNet, a tool used for miRNA-target interaction and pathway enrichment analysis.

has-miR-29a-3p, has-miR-132-3p, has-miR-25-3p and hsa-miR-92a-3p.

KEGG Pathways	Hits	adj. Pval
Pathways in cancer	144	4.54e-13
Cell cycle	73	7.25e-13
Small cell lung cancer	47	3.01e-8
Prostate cancer	49	7.28e-8
**Focal adhesion**	**90**	**1.33e-7**
HTLV-I infection	89	1.97e-7
Chronic myeloid leukemia	41	9.29e-7
p53 signaling pathway	38	0.00000296
Pancreatic cancer	38	0.00000439
**Neurotrophin signaling pathway**	**57**	**0.0000134**
**Protein processing in endoplasmic reticulum**	**58**	**0.0000331**
Glioma	34	0.0000629
Colorectal cancer	27	0.000147
MAPK signaling pathway	99	0.000301
Bacterial invasion of epithelial cells	29	0.000301
**Adherens junction**	**34**	**0.000336**
TGF-beta signaling pathway	39	0.000336
**Regulation of actin cytoskeleton**	**72**	**0.000336**
ErbB signaling pathway	40	0.000336
Melanoma	33	0.000358
Non-small cell lung cancer	27	0.000358
Renal cell carcinoma	30	0.000358
Epstein-Barr virus infection	41	0.000399
Progesterone-mediated oocyte maturation	37	0.000425
ECM-receptor interaction	38	0.000592

Supplementary Table S1. Cont.

KEGG Pathways	Hits	adj. Pval
Wnt signaling pathway	58	0.0007
**RNA transport**	**52**	**0.0007**
Hepatitis C	43	0.000821
Bladder cancer	17	0.000876
Oocyte meiosis	45	0.00131
Chagas disease (American trypanosomiasis)	38	0.002
Endometrial cancer	22	0.00216
Salmonella infection	32	0.00216
Lysine degradation	23	0.00225
Shigellosis	23	0.00225
Influenza A	43	0.00356
Toxoplasmosis	38	0.00454
Acute myeloid leukemia	25	0.00871
Insulin signaling pathway	51	0.00877
**Apoptosis**	**33**	**0.0137**
Thyroid cancer	14	0.016
mTOR signaling pathway	20	0.0164
Fc gamma R-mediated phagocytosis	37	0.0175
Notch signaling pathway	20	0.0275
Citrate cycle (TCA cycle)	14	0.0304
Circadian rhythm - mammal	11	0.0337
**Dopaminergic synapse**	**44**	**0.0343**
Long-term potentiation	27	0.0367
Ribosome biogenesis in eukaryotes	22	0.0394
Aldosterone-regulated sodium reabsorption	15	0.0394

Supplementary Table S1. Cont.

KEGG Pathways	Hits	adj. Pval
NOD-like receptor signaling pathway	20	0.0394
Viral myocarditis	12	0.0465
Pathogenic Escherichia coli infection	15	0.0494
Herpes simplex infection	36	0.0646
Epithelial cell signaling in Helicobacter pylori infection	15	0.0769
Axon guidance	40	0.0769
Cysteine and methionine metabolism	14	0.0769
Huntington's disease	12	0.0769
Gap junction	31	0.0847
RNA degradation	22	0.0887
Endocytosis	34	0.107
Dorso-ventral axis formation	6	0.111
Arrhythmogenic right ventricular cardiomyopathy (ARVC)	6	0.111
RIG-I-like receptor signaling pathway	18	0.115
Alzheimer's disease	18	0.115
mRNA surveillance pathway	28	0.115
Aminoacyl-tRNA biosynthesis	4	0.127
Hypertrophic cardiomyopathy (HCM)	10	0.146
Pyruvate metabolism	15	0.146
Glycosaminoglycan biosynthesis - chondroitin sulfate	6	0.146
Cholinergic synapse	31	0.152
Measles	33	0.153
Tight junction	37	0.186
Long-term depression	23	0.192
T cell receptor signaling pathway	31	0.199

Supplementary Table S1. Cont.

KEGG Pathways	Hits	adj. Pval
Prion diseases	8	0.221
B cell receptor signaling pathway	24	0.221
Thiamine metabolism	2	0.223
Synaptic vesicle cycle	7	0.223
Selenocompound metabolism	5	0.238
Pertussis	17	0.238
Synthesis and degradation of ketone bodies	4	0.238
Alanine, aspartate and glutamate metabolism	11	0.246
Arginine and proline metabolism	18	0.246
Vibrio cholerae infection	7	0.253
Transcriptional misregulation in cancer	7	0.253
Jak-STAT signaling pathway	30	0.257
Endocrine and other factor-regulated calcium reabsorption	12	0.257
Cocaine addiction	14	0.258
Phosphatidylinositol signaling system	23	0.269
Valine, leucine and isoleucine degradation	14	0.286
VEGF signaling pathway	23	0.288
Type II diabetes mellitus	15	0.296
Glycosylphosphatidylinositol (GPI)-anchor biosynthesis	6	0.301
Serotonergic synapse	26	0.309
Hedgehog signaling pathway	17	0.315
Dilated cardiomyopathy	23	0.324
Amphetamine addiction	19	0.332
Fc epsilon RI signaling pathway	22	0.333
Leukocyte transendothelial migration	31	0.336

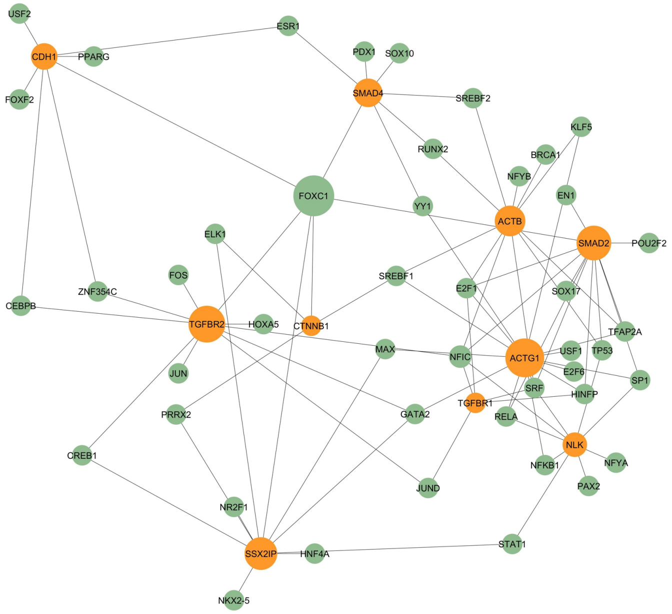

(A) **Adherens junction**

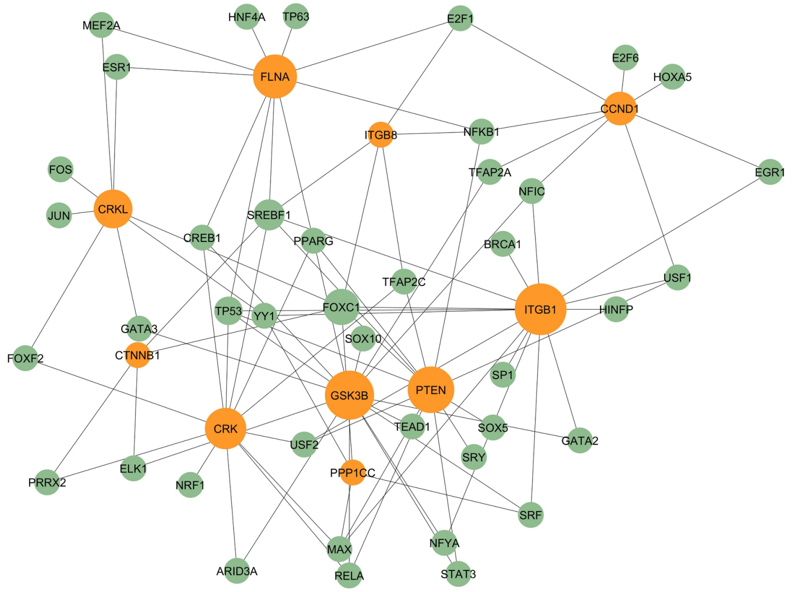

(B) **Focal Adhesion**

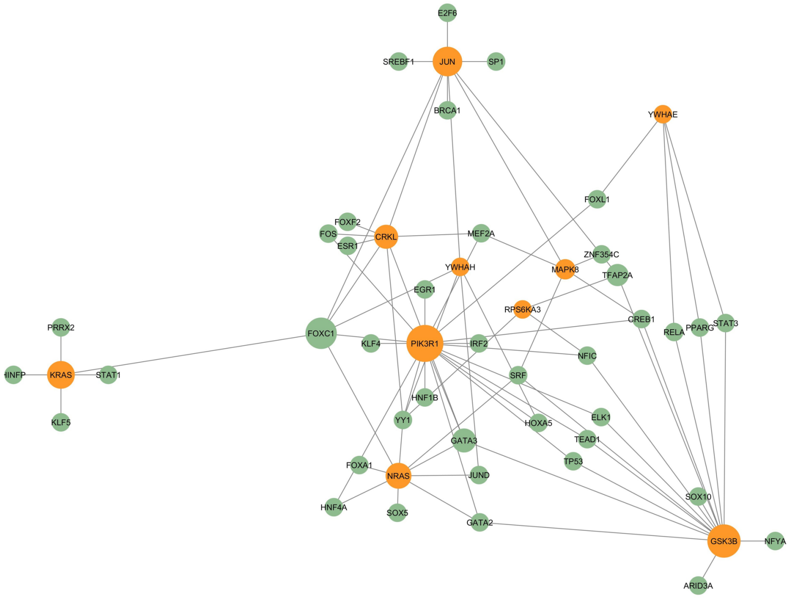

(**C**) Neurotrophin signaling pathway

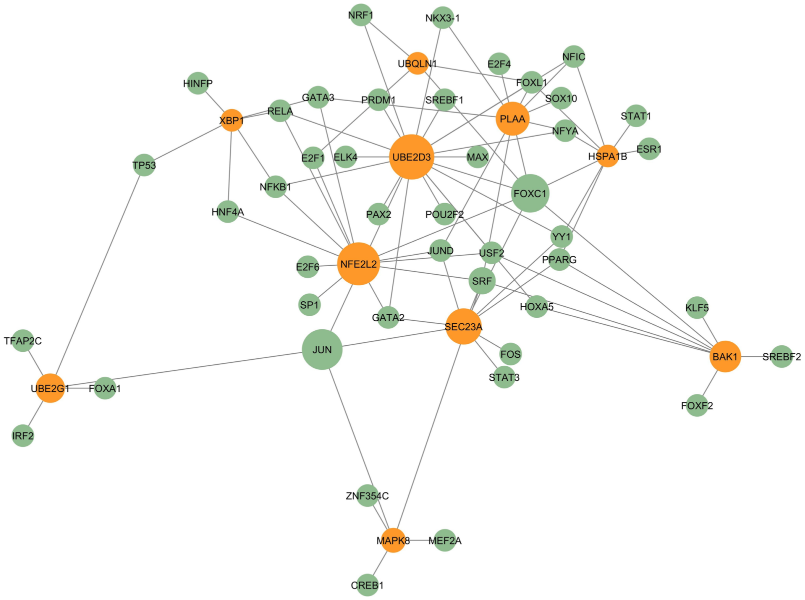

(**D**) Protein processing in endoplasmic reticulum

Supplementary Figure S1. Transcription factor-gene interaction networks. The interaction networks show relationships between hub genes of KEGG pathways (orange nodes) and transcription factors (green nodes). The most important nodes in the network are represented as larger sizes, derived from the Betweenness Centrality Score. The distribution of the nodes is arranged in a way that reflects the proximity of their interactions, with nodes that are closely related or interact more frequently placed closer together. Panel A shows Adherens junctions, Panel B shows Focal adhesion, Panel C shows the Neurotrophin signaling pathway, and Panel D shows Protein processing in the endoplasmic reticulum pathways.

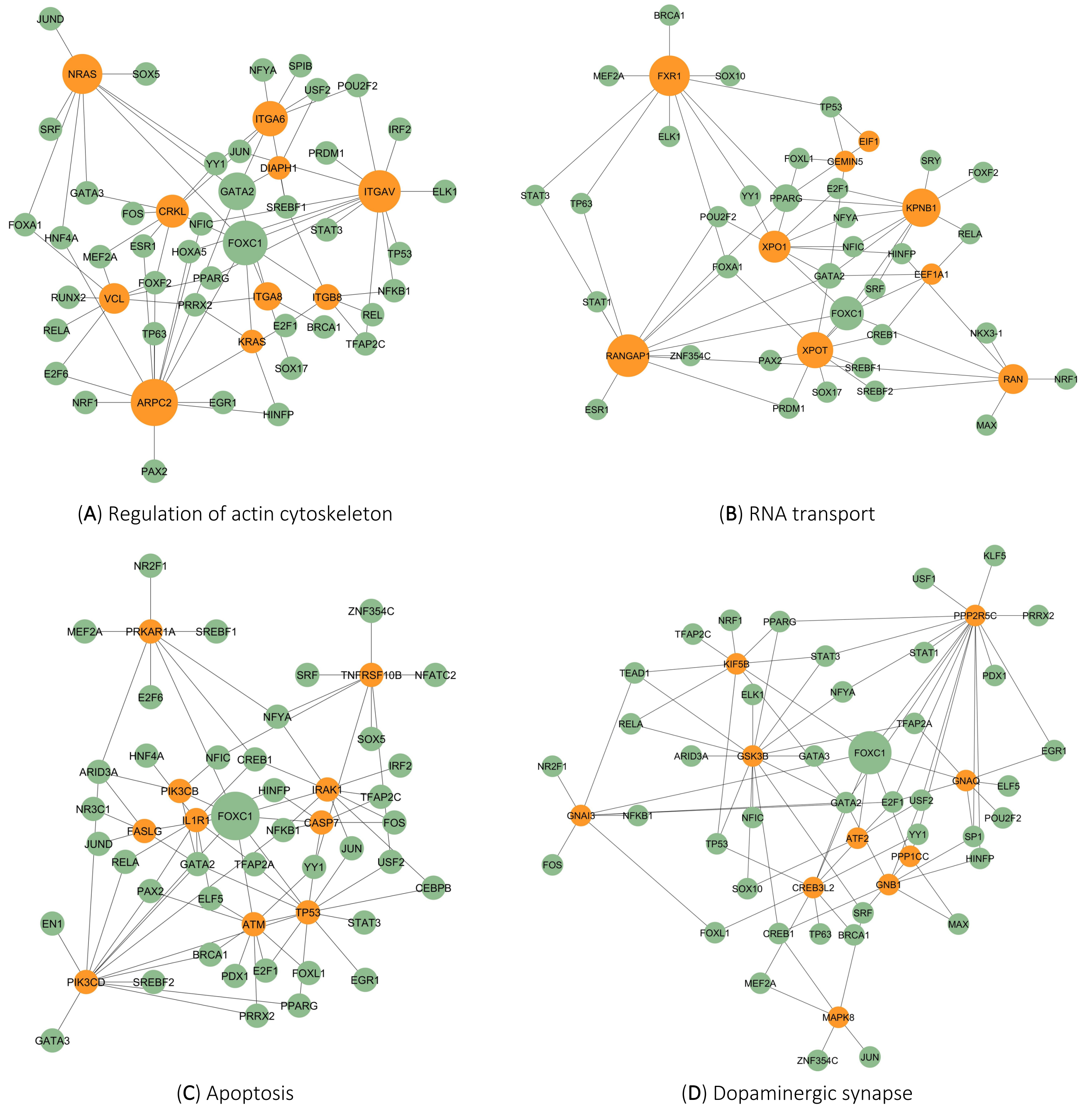

Supplementary Figure S2. Transcription factor-gene interaction networks. The interaction networks show relationships between hub genes of KEGG pathways (orange nodes) and transcription factors (green nodes). The most important nodes in the network are represented as larger sizes, derived from the Betweenness Centrality Score. The distribution of the nodes is arranged in a way that reflects the proximity of their interactions, with nodes that are closely related or interact more frequently placed closer together. Panel A shows the actin cytoskeleton, Panel B shows the RNA transport pathway, Panel C shows the apoptosis pathway, and Panel D shows the dopaminergic synapse pathway.
